# An algorithm for automated layout of process description maps drawn in SBGN

**DOI:** 10.1093/bioinformatics/btv516

**Published:** 2015-09-10

**Authors:** Begum Genc, Ugur Dogrusoz

**Affiliations:** ^1^The Insight Centre for Data Analytics, University College Cork, Western Road, Cork, Ireland,; ^2^Computer Engineering Department, Faculty of Engineering, Bilkent University, Ankara 06800, Turkey and; ^3^Sander Lab, Memorial Sloan-Kettering Cancer Center, 417 E68th St., New York, NY 10065, USA

## Abstract

**Motivation:** Evolving technology has increased the focus on genomics. The combination of today’s advanced techniques with decades of molecular biology research has yielded huge amounts of pathway data. A standard, named the Systems Biology Graphical Notation (SBGN), was recently introduced to allow scientists to represent biological pathways in an unambiguous, easy-to-understand and efficient manner. Although there are a number of automated layout algorithms for various types of biological networks, currently none specialize on process description (PD) maps as defined by SBGN.

**Results:** We propose a new automated layout algorithm for PD maps drawn in SBGN. Our algorithm is based on a force-directed automated layout algorithm called Compound Spring Embedder (CoSE). On top of the existing force scheme, additional heuristics employing new types of forces and movement rules are defined to address SBGN-specific rules. Our algorithm is the only automatic layout algorithm that properly addresses all SBGN rules for drawing PD maps, including placement of substrates and products of process nodes on opposite sides, compact tiling of members of molecular complexes and extensively making use of nested structures (compound nodes) to properly draw cellular locations and molecular complex structures. As demonstrated experimentally, the algorithm results in significant improvements over use of a generic layout algorithm such as CoSE in addressing SBGN rules on top of commonly accepted graph drawing criteria.

**Availability and implementation:** An implementation of our algorithm in Java is available within ChiLay library (https://github.com/iVis-at-Bilkent/chilay).

**Contact:** ugur@cs.bilkent.edu.tr or dogrusoz@cbio.mskcc.org

**Supplementary information:** Supplementary data are available at *Bioinformatics* online.

## 1 Introduction

Popular belief is that diagrams directly address people’s innate cognitive abilities (Larkin and Simon, 1987). Due to the fact that symbols, diagrams and other graphical representations vary widely around the world, it is necessary to have a common interpretation. Standard notations play an important role in communication and facilitate rapid development in many research areas.

To address this issue in the field of systems biology, a group of modelers, biochemists and software engineers published the Systems Biology Graphical Notation (SBGN), which allows scientists to represent biological pathways and networks in an easy-to-understand and efficient way (Le Novère *et* *al.*, 2009). It consists of three complementary languages: process descriptions (PD), activity flows and entity relationships.

**Fig. 1.**
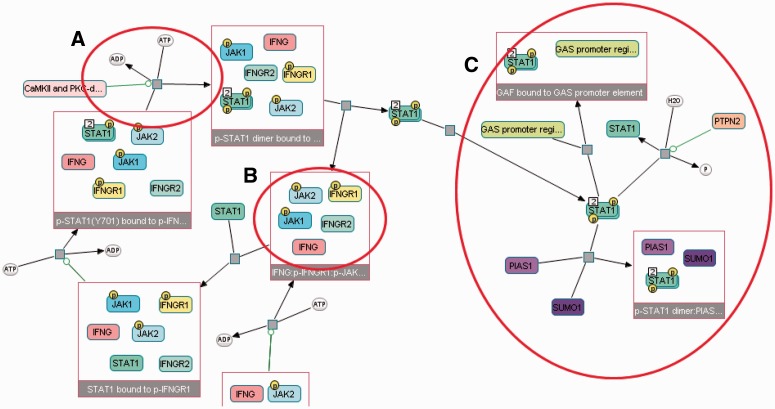
SBGN states that product and substrate edges of a process node (small gray squares) should be placed on opposite sides of the associated process node, attached via an input and an output port, respectively (**A**). A general purpose layout algorithm will not properly pack degree 0 members (rounded rectangles with information bulbs) inside a molecular complex (**B**). The processes that take place inside a cellular compartment are not clearly separated from those occurring outside that compartment (**C**)

In this article, we propose a new automated layout algorithm that enforces SBGN-specific rules for PD maps. As depicted in Figure 1, layouts produced by general purpose graph layout algorithms fall short in certain significant ways:

- Product and substrate edges of a process node are not necessarily placed on opposite sides of associated process nodes. Moreover, SBGN states that each process has two ports as attachment points.
- Degree zero members inside a molecular complex are not efficiently packed, often wasting large amounts of area.
- Cellular locations of processes are not shown in the map.

Our proposed layout algorithm is the only one that successfully addresses these issues, producing layouts that comply with SBGN-PD notation. Other software providing SBGN-PD maps make use of generic layout algorithms with limited success. For instance, Vanted (Junker *et* *al.*, 2006) provides generic force-directed layout with no support for compound structures. CellDesigner (Funahashi *et* *al.*, 2003) also provides a rich set of generic layout algorithms, including one with compound support imported from a commercial library.

## 2 Background

### 2.1 Graphs and PD maps

**Fig. 2.**
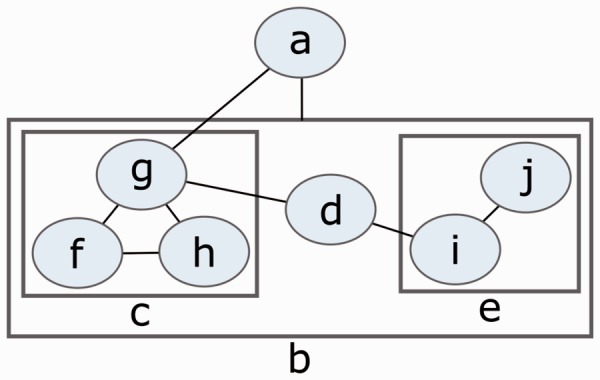
An example compound graph of multiple levels of nesting, where V={a,b,…,j}, E={{a,b},{a,g},{d,e},{d,g},{f,g},{f,h},{g,h},{i,j}} and F={bc,bd,be,cf,cg,ch,ei,ej}

The basics of graph theory are provided in the Supplementary Material. A *c*ompound graph (Fig. 2) *C* = (*V*, *E*, *F*) consists of nodes *V*, adjacency edges *E* and inclusion edges *F* (Dogrusoz *et* *al.*, 2009).

**Fig. 3.**
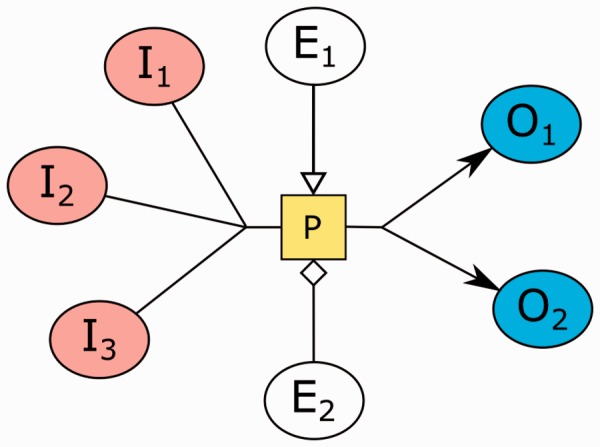
A sample process node with three subtsrate nodes and two product nodes, and two effector (one modulator and one stimulator) nodes

An SBGN-PD map represents all the molecular processes and interactions taking place between biochemical entities, and their results. The underlying representation is essentially a bipartite compound graph. These maps depict how entities or so-called *entity pool nodes* (EPN) transition from one form to another as a result of different influences, portraying the temporal qualities of molecular events occurring in biochemical reactions (Le Novère *et* *al.*, 2009). The way in which one type of entity is transformed into another is conveyed by a *process node.* We call EPN’s consumed and produced by a process *substrate* (input) and *product* (output) nodes, respectively. In addition, the EPNs that control (e.g. modulate or stimulate) a process are called *effector nodes* (Fig. 3).

An exchange file format for SBGN maps named SBGN-ML was recently introduced (van Iersel *et* *al.*, 2012).

### 2.2 Automated layout and Compound Spring Embedder (CoSE)

The purpose of performing layout on a graph is to make a pictorial representation that is as clear and pleasant as possible. A poor layout of a graph may confuse the user, whereas a well-organized and aesthetically pleasing layout can improve the user’s ability to understand the underlying data. Criteria of a good layout may differ from person-to-person. However, among the generally accepted ones (Battista *et* *al.*, 1998) are minimal total drawing area, number of edge–edge crossings and total edge length, producing uniform edge lengths, and ability to reflect any symmetries in the network.

Force-directed layout algorithms (also known as spring embedders) are arguably the most popular type of automatic graph layout, where the basic idea is to simulate a physical system obeying the laws of Hooke and Coulomb.

CoSE is a force-directed layout algorithm that supports compound nodes (Dogrusoz *et* *al.*, 2009). Certain additions have been made on the basic spring embedder model to handle compound nodes. The main idea is to represent an expanded node and its associated nested graph as a single entity, similar to a ‘cart’, which can move freely (details provided in the Supplementary Material).

### 2.3 Rectangle packing and compaction

The rectangle packing problem can be defined as packing a number of non-uniformly sized, rectangle-shaped objects into a container, such that there will be no overlaps between the objects and the container will be as compact as possible. This problem, defined in two-dimensions, is an NP-hard problem (Garey and Johnson, 1979).

Almost all graph drawing algorithms try to minimize drawing area by assuming that the graph is connected (Dogrusoz *et* *al.*, 2002). However, if the graphs have disconnected members (e.g. members of molecular complexes), most such algorithms yield poor results in respect to minimizing wasted area. Various packing techniques have been used in graph layout to pack disconnected parts (disconnected nodes or connected components) of a graph, including tiling (Dogrusoz, 2002) and polyomino packing (Freivalds *et* *al.*, 2001). Success of a packing method is usually measured by the *adjusted fullness* of the resulting drawing, which is basically the ratio of the total area of the nodes being packed to the area of the tightest bounding rectangle with specified aspect ratio for the drawing.

Results of packing could often be improved through computation of a visibility graph and applying compaction (de Berg *et* *al.*, 2008). The *visibility* in this context refers to the feasibility of drawing a collision-free straight line between two nodes.

## 3 Methods

We introduce a new, specialized algorithm for layout of SBGN-PD maps. Since SBGN-PD notation makes exclusive use of compound structures, our algorithm was based on CoSE, addressing SBGN-specific rules in PD maps as summarized here and detailed subsequently:

- Additional force types and associated procedures on top of the force scheme employed by CoSE were introduced to congregate substrate and product edges at input and output process ports, respectively, and to place substrate and product entities on opposite sides of the associated process.
- Tiling or other packing methods are employed to produce more compact and aesthetically pleasing layouts of disconnected nodes.
- Display of cellular locations is no longer an issue since CoSE can handle any level of nesting.

### 3.1 Handling process nodes

**Fig. 4.**
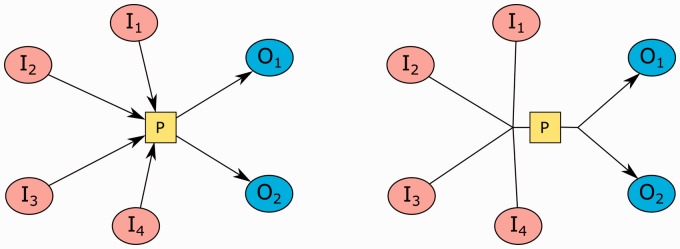
Drawing of processes in CoSE (left) versus SBGN (right); SBGN makes use of ports to clearly separate what is consumed and produced by a process

SBGN rules state that substrates and products of a process can only attach to the process from its input and output ports, respectively, placed vertically or horizontally on opposite sides. In order to avoid unnecessary edge crossings and clearly display the flow in a process, its substrate and product nodes should be positioned near the associated ports. Besides, not clearly separating substrates and products of a process via ports will make reversible processes ambiguous. However, generic layout algorithms, including CoSE, will not respect this convention (Fig. 4).

#### 3.1.1 Handling process ports

**Fig. 5.**
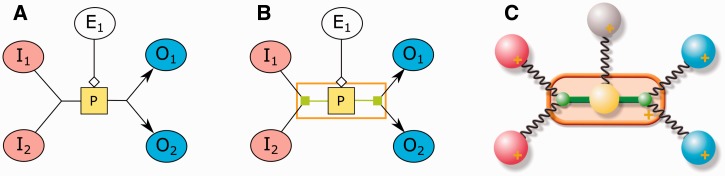
How a process with two substrates, two products and one effector node should be displayed in an SBGN-PD map (**A**). How our algorithm internally represents such a process using newly introduced dummy port nodes (small filled square) and edges, and process container compound node (unfilled rectangle) (**B**). Associated physical model of our algorithm (gravitational fields not shown for brevity) (**C**)

In order to equip process nodes with ports in SBGN-PD layout, without interfering with the existing physical system too much, we introduce new node and edge types (Fig. 5). A new node type called *port node* is introduced to represent ports of a process. These nodes are set to have negligible dimensions. In addition, a new edge type called *port edge* is introduced to keep a process node and its two associated port nodes together. These edges are assumed to be ‘rigid’, not exerting any spring forces on the associated port and process nodes. Finally, a new compound node type called *process container* is introduced to enclose and tightly keep together the associated process along with the newly introduced dummy port nodes and edges.

We treat these new node and edge types specially, and do not calculate spring forces for rigid edges, repulsion forces between a process and its port nodes, and gravitation forces for process and port nodes.

#### 3.1.2 Orienting processes

**Fig. 6.**
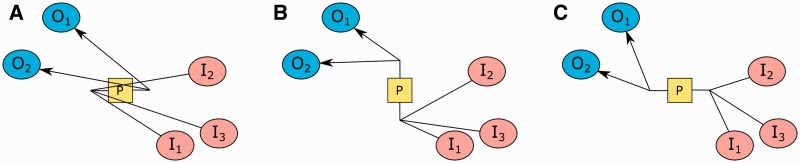
Illustration of how the orientation of a process might affect layout: original orientation is left-to-right by default (**A**), left rotation by 90° (bottom-to-top) eliminates self crossings (**B**), and another left rotation by 90° (right-to-left) further improves layout (**C**)

SBGN allows ports of a process node to be placed either horizontally or vertically. Without proper orientation of substrates and products of a process, layout might easily produce edge crossings even with a single process (Fig. 6). To avoid such problems and properly orient processes and place their substrates (products) near the input (output) port, we introduce a new force type named *rotational force* into the force scheme.

Rotational forces are exerted on dummy container nodes by the associated substrate and product nodes, which indirectly results in rotation of process nodes. The main idea behind applying rotation is that if a group of neighboring nodes persistently pull their process node in a direction against the current orientation of the process, a decision is made to change the orientation of the container compound node by either applying a 90° or 180° rotation. In SBGN, a process node may assume one of four discrete orientations: left-to-right, right-to-left, bottom-to-top and top-to-bottom.

**Fig. 7.**
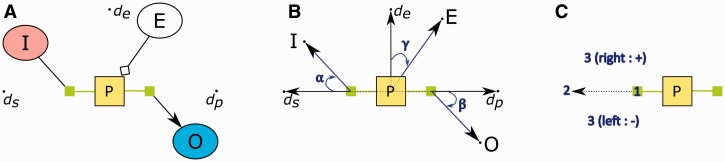
Rotational force acting on process *P* is calculated using the angles neighboring nodes make with their connection points. A sample process with three neighbors, where *d*_p_ (*d*_s_ or *d*_e_) represents the desired location of an input node (output node or effector node) (**A**). The angles that these neighbors make with the process node with respect to the current orientation (**B**). Illustration of how the signs of these angels are calculated using the left-turn rule. In this example, a left turn is assumed to signify a negative sign (**C**)

The magnitude of the rotational force *F*_t_(*P*) acting on a process node *P* should be proportional to how much the neighboring nodes deviate from their ideal positions:

(1) $$||F_{t}(P)||=\sum_{i=1}^{n_{\text{s}}}\alpha_{i}+\sum_{i=1}^{n_{\text{p}}}\beta_{i}+\sum_{i=1}^{n_{\text{e}}}\gamma_{i},$$

where *n*_s_ (*n*_p_ or *n*_e_) denotes the number of substrate (product or effector) nodes of process *P*, and *α_i_* (*β_i_* or *γ_i_*) denotes the angle *i*th substrate (product or effector) node makes with the line ray emanating from the center of the process node and going toward the input port (output port or ideal effector position) (Fig. 7B).

Absolute value of these angles could be calculated by taking cross product between the vectors from the connection point (associated port or center of process) to the neighboring node location and from the connection point to the ideal position of the neighboring node. For instance, for substrate *i*:

(2) $$|\alpha_{i}|=\arccos\frac{\vec{I_{i,x}}\cdot \vec{d_{s,x}}+\vec{I_{i,y}}\cdot \vec{d_{s,y}}}{\left||\vec{I_{i}}||\cdot ||\vec{d_{s}}|\right|},$$

where $\vec{I_{i}}$ is the vector from the input port to the current location of substrate *i*, with respective horizontal and vertical components $\vec{I_{i,x}}$ and $\vec{I_{i,y}}$, and $\vec{d_{s}}$ is the vector from the input port to the ideal location of substrate *i.* The calculation is similar for products and effectors.

Notice, however, that the signs of these angles should be taken into account. This could be easily calculated by performing a *left test.* If the expression

(3) $$\left(x_{2}-x_{1})(y_{3}-y_{1})-(y_{2}-y_{1})(x_{3}-x_{1}\right)$$

is >0, it means a left turn was taken, where (*x*_2_, *y*_2_), (*x*_1_, *y*_1_) and (*x*_3_, *y*_3_), respectively, specify the location of the neighboring node, the connection point (associated port or the process center) and the ideal position. A left turn and a right one must signify opposite signs as exemplified in Figure 7C.

If the net rotational force $F_{t}(P)$ acting on a process node *P* exceeds a predefined threshold determined empirically, the process is rotated by 90° in clockwise or counter-clockwise direction, depending on its sign.

**Fig. 8.**
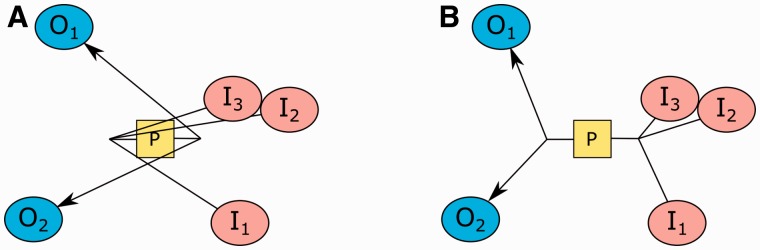
An example, where a rotation is needed but not detected by the heuristic used for 90° rotations defined earlier (**A**). The same process, after its ports are swapped via a 180° rotation (**B**)

In certain cases, however, this heuristic will not suggest any rotations even though a rotation is strongly needed (Fig. 8). In fact, in such cases, a 180° rotation or a swap operation is likely to drastically improve the situation. One can determine such cases by simply checking whether or not a majority of neighboring nodes have obtuse angles as defined earlier. This check is performed before the 90° rotation case since it is more drastic, yielding more improvement. Again, what proportion of the neighboring nodes constitutes a ‘majority’ is determined empirically.

Rotational forces are summed up for a number of iterations. Once every pre-defined fixed number of iterations, the sum is normalized and each process node is checked for whether or not a swap or a rotation is needed. For the sake of stability, only one swap or rotation is allowed even when multiple processes qualify.

#### 3.1.3 Gathering substrates and products

Proper orientation of processes will only be possible if any multiple substrates (products) are placed near each other. We make use of an additional location enhancement heuristic for this purpose.

**Fig. 9.**
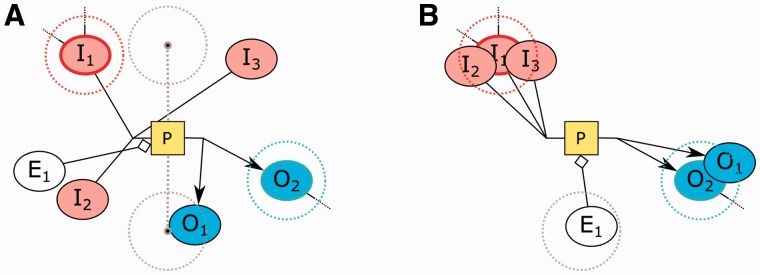
An example, where locations of substrate, product and effector nodes (**A**) are enhanced using our heuristic (**B**). Higher degree nodes *I*_1_ and *O*_2_ are chosen as seeds for substrate and product nodes, respectively. The effector *E*_1_ is closer to the bottom of the two ideal positions for the left-to-right process *P* it is associated with

The idea is not to interfere with the placement of ‘hop’ nodes that are of degree 2 or higher but bring any degree 1 nodes, which are ‘free’ to move without affecting the overall structure of the spring system, closer to such high degree nodes. This should not only help with satisfying the SBGN-PD convention with respect to properly gathering substrates (products) together but also speed up convergence. Consequently, we periodically identify a substrate (product) node with highest degree and place any degree 1 substrate (product) node near it. In order to avoid any extreme amounts of repulsion forces and exploit the power of randomization, we place degree 1 nodes randomly within a circle centered at this highest degree node (Fig. 9).

Ideally, an effector should be placed in between products and substrates. The location enhancement heuristic is similarly performed on effector nodes to avoid an effector node getting ‘stuck’ among product (substrate) nodes. Consequently, once in a while, we pull all effector nodes near their ideal position. Notice, however, that for each orientation of a process, there will be exactly two ideal locations for effectors. For instance, for a process oriented from left-to-right, these are vertically aligned with the center of the process, one being on top of the process, and the other on the bottom, both separated from the process by ideal edge length. Again, we opt to apply a minimal amount of randomness rather than placing them on the exact ideal position for the same reasons explained earlier (Fig. 9).

#### 3.1.4 Modifications on CoSE

In order to properly handle process nodes and satisfy SBGN-specific constraints on them, we first add a new, second phase to the CoSE algorithm, and decrease the number of iterations CoSE performs in the first phase since a ‘draft’ layout should suffice. Starting with this draft layout, the new phase is responsible for addressing SBGN rules without ruining the resultant layout, which is assumed to satisfy generally accepted layout criteria. The difference between the SBGN phase and earlier one can be summarized as follows. Since the SBGN phase is expected to make local changes in the layout, the system starts out from a lower cooling factor. Rotational forces are calculated for each process node on top of the spring, repulsion and gravitational forces calculated by CoSE. To represent process nodes and their ports with newly introduced dummy nodes, associated CoSE method needs to be expanded as in Algorithm 1. Hence, the convergence is no longer solely dependent on node movements going below a certain threshold. We also try to ensure that all substrates and products of every process are properly oriented (Algorithm 2).

**Algorithm 1** Moving nodes and applying rotation

1: **function** calcNodePositionsAndSizes(*C*)

2:  rotationList←∅ //candidates for rotation

3:  **for all** process node P∈V(C) with ports *p_i_* and *p_o_* **do**

4:   TransferForces(*P*)

5:   ResetForces(*P*, *p_i_*, *p_o_*)

6:  **end** **for**

7:  **if **(phase=SBGN)∧(totalIter%rotPeriod=0) **then**

8:   **for all** process node P∈V(C) **do**

9:     **if** NeedsRotation(*P*) **then**

10:      rotationList.add(P)

11:     **end** **if**

12:   **end** **for**

13:   RotateRandomOne(*rotationList*)

14:  **end** **if**

15:  CoSE.calcNodePositionsAndSizes(*C*)

16: **end** **function**

**Algorithm 2** New second (SBGN-PD) phase

1: **function** doPhase2(*C*)

2:  totalIterations←0

3:  $initialCoolingFac\leftarrow c_{cool}$ //start cooler

4:  **while** *totalIter* < *maxIter* **do**

5:   totalIter←totalIter+1

6:   **if **totalIter % apprPeriod=0 **then**

7:     approximateLocations()

8:   **end** **if**

9:   **if** converged() ∧ edgesProperOriented() **then**

10:     **break**

11:   **end** **if**

12:   UpdateBounds(*C*) //resize compounds

13:   calcSpringForces()

14:   calcRepulsionForces()

15:   calcGravitationalForces()

16:   moveNodes() //move nodes based on total forces

17:  **end** **while**

18: **end** **function**

### 3.2 Packing disconnected nodes

Disconnected nodes come up quite frequently in SBGN-PD diagrams, especially with molecular complexes, where members of a molecular complex are all degree zero. In fact, a molecular complex might be recursively defined from another one, resulting in potentially arbitrary levels of nested disconnected nodes. Thus, any algorithm to tightly pack molecular complex members could work bottom-up, and could be easily implemented recursively. Disconnected nodes outside molecular complexes, on the other hand, are highly unlikely but not impossible to come across.

This problem is a special case of the popular rectangle packing problem discussed earlier. Various techniques such as tiling and polyomino packing have been used in the past to solve this problem in the context of graph drawing. Since polyomino packing results are superior only with larger number of nodes, as will be shown later on, we went with tiling due to its simplicity for implementation and faster running time. Notice that, most of the time, the number of rectangles to be packed is only a few and all with similar dimensions. Hence, use of a complicated algorithm is unlikely to produce significantly more compact drawings.

Packing can be integrated into SBGN-PD layout without interference as a pre-processing step as explained in the Supplementary Material. We would also like to remark that packing should only be applied to a compound node with no edges (intra-graph or inter-graph edges) in it. Any non-degree 0 node contained in a compound structure should not be forced to a location determined by a packing algorithm but rather should be free to move near its neighbor(s).

#### 3.2.1 Further compaction

After application of a packing algorithm, it is common to have more room for improvement, which can be achieved by calculating the visibility graph of the disconnected set of nodes to be packed. A visibility graph in a certain orientation, for example bottom-to-top, is a *directed acyclic graph* and represents the visibility of each node when ‘looked’ from that node vertically toward the up direction. We say that node *v* is visible by node *u* in bottom-to-top direction if *v* is above *u*, and *u* can completely ‘see’ node *v* with no obstruction in between two nodes, looking from bottom-to-top. In other words, the nodes directly below, to the right or to the left of a node *u,* are not visible by *u.* By using the directed acyclic structure of visibility graphs, a topological sort is applied to get the objects in order, and one-by-one in the computed topological order, each object is moved closer to its ascendant.

Even though application of this algorithm in either one of four directions might produce more compact drawings, the improvements are usually minimal if any. Also notice that a separate calculation of the visibilities is required for each direction.

### 3.3 Running time

CoSE algorithm runs in $O(k\cdot (|V{|}^{2}+|E|))$ time, where the underlying compound graph is represented with C=(V,E,F), and *k* is the number of iterations needed to converge. This is due to the simple fact that, in each iteration, repulsion forces are calculated between each node pair and spring forces are calculated for each edge. Additional heuristics employed by our algorithm do not increase the asymptotic running time since rotational forces are calculated for each process node. Similarly, packing is linear in the number of nodes to be packed, which is at most as many as all the nodes in the compound graph. Our experiments as described in the following section confirm this theoretical running time analysis.

## 4 Implementation and Results

We implemented SBGN-PD layout within an open source layout library called Chisio Layout (ChiLay). The experiments outlined below were performed on an ordinary PC (with Intel® Core^TM^ i7-4600U 2.10 GHz processor, 8 GB RAM, and 256 GB SSD). For each measurement for a layout algorithm, 10 executions were performed and the average was taken since spring embedders start out from random initial positions, and this might highly affect the convergence speed.

### 4.1 Packing

For comparing tiling and polyomino packing methods, random compound graphs with no edges were generated. Details of these can be found in the Supplementary material. Further compaction through visibility is usually of no use with tiling. More importantly, as can be seen from the results, polyomino packing has a clear advantage over tiling with large number of nodes (>60) but for smaller graphs, like SBGN-PD maps, tiling performs just as well.

As our tests confirm, tiling is significantly faster than polyomino packing. However, since SBGN maps have relatively small number of nodes, running time spent on packing is negligible.

### 4.2 Parameter tuning

Our layout algorithm has a number of parameters to customize its behavior. We tested the behavior of our algorithm with respect to each such parameter and applied a comprehensive test to fine-tune it.

In order to perform these experiments, we used 34 ‘real-life’ SBGN-PD maps as taken from Pathway Commons (Cerami *et* *al.*, 2011) database, using querying and conversion (to SBGN-ML) facilities of Paxtools (Demir *et* *al.*, 2013). These maps were chosen to be of varying types including regulation and signaling networks, not larger than a few hundred nodes. For larger graphs, at least one complexity management technique can be used (Dogrusoz and Genc, 2006).

The main criterion used for the success of the algorithm is the ratio of ‘properly oriented’ edges to total number of edges in the graph. To decide when an edge is properly oriented, we use a parameter named *angle tolerance* (*at*). Other parameters of our algorithm are *approximation distance* (*ad*), *approximation period* (*ap*), *rotation period* (*rp*), *90-degree rotation force threshold* (c90), *180-degree rotation ratio threshold* *(c180)* and *phase 1 maximum iteration count* (*ip*1).

Before experimenting with individual parameters, we wanted to find the most coherent set of values of these parameters given a discrete set of values for each parameter as specified earlier. The best results are obtained when ad=50, ap=211,rp=2, c90=70, c180=0.5, and ip1=200. To confirm that changes in these parameters do not interfere with each other, we performed tests where only one parameter at a time was changed. The results can be found in the Supplementary Material along with other details.

### 4.3 Comparison with CoSE

**Fig. 10.**
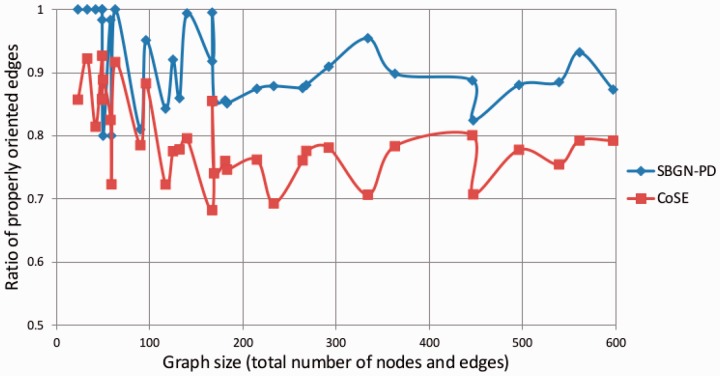
Comparison of the success of our algorithm with CoSE (graph size versus ratio of properly oriented substrate, product and effector edges)

We have compared the success rate of our algorithm in properly orienting edges with the generic algorithm CoSE. As Figure 10 shows, there is a clear advantage of using the extra heuristics.

**Fig. 11.**
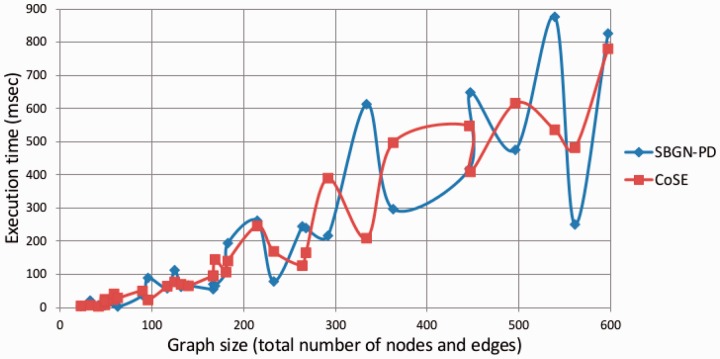
Comparison of the running time of our algorithm with CoSE (graph size versus execution time in milliseconds)

In terms of execution time, our algorithm performs as well as CoSE (Fig. 11). Actually, as the iteration count required to complete phase 2 increases, the number of rotation operations needed by our algorithm increases as well. However, since our algorithm applies tiling to disconnected nodes and ignores such nodes during layout, the decreased graph size seems to compensate for the extra time used by newly introduced heuristics.

We also investigated whether or not the behavior of our algorithm depends on graphs being simple or not. Our experiments show that when there are no compound structures in the graph, ratio of properly oriented edges goes up even further to around 95%.

**Fig. 12.**
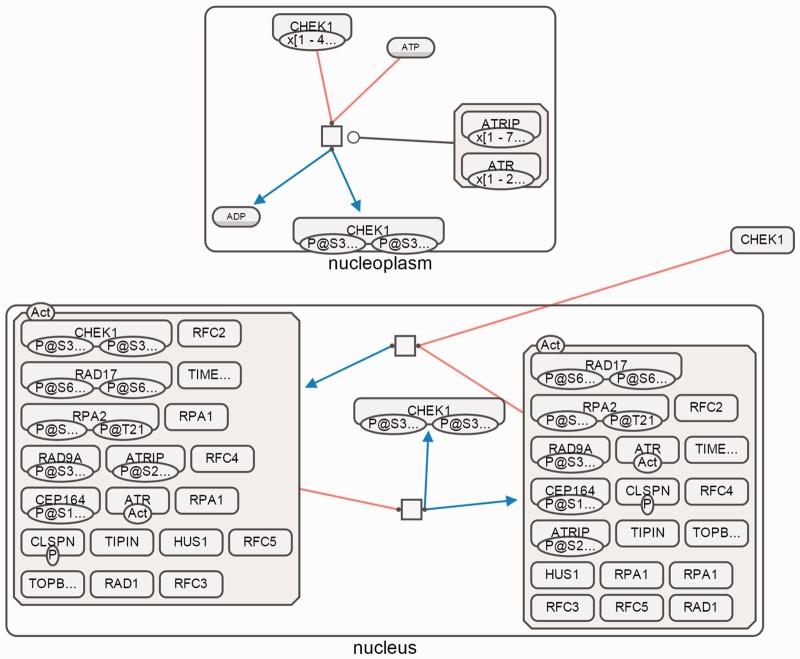
Paths between ATRIP and CHEK1 as laid out by our algorithm

**Fig. 13.**
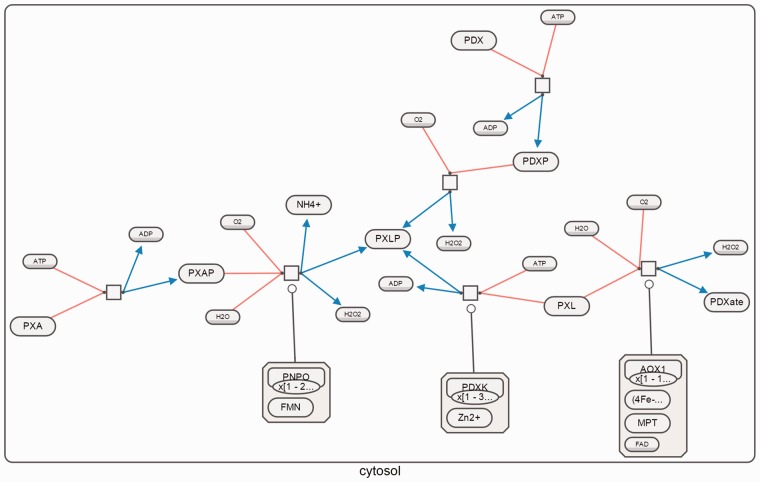
Vitamins B6 activation to pyridoxal phosphate as laid out by our algorithm; all edges were properly oriented

**Fig. 14.**
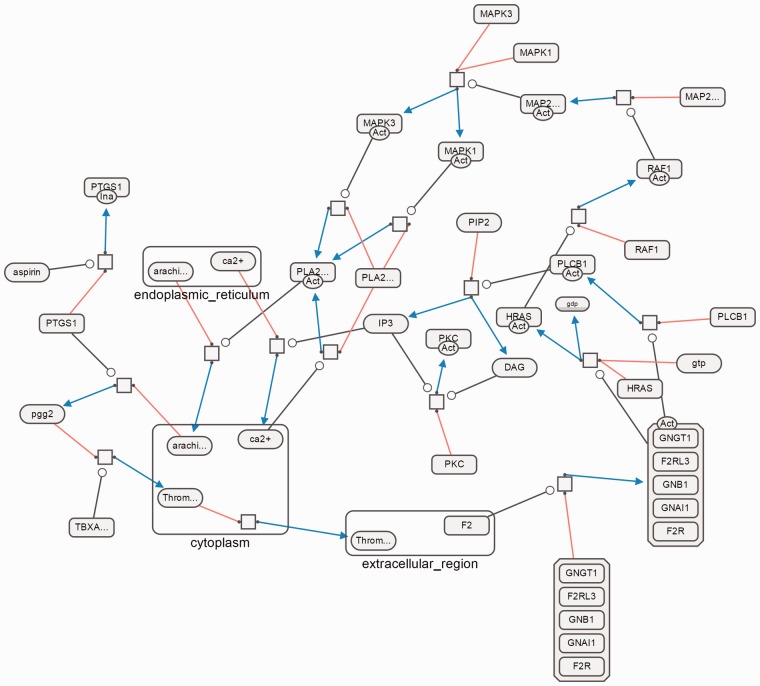
Aspirin blocks signaling pathway involved in platelet activation as laid out by our algorithm

Figures 12, 13 and 14 show sample SBGN-PD maps laid out using our algorithm using SBGNViz, which is a specialized visualization tool developed for SBGN PD maps (Sari *et* *al.*, 2015). More examples are available in the Supplementary Material.

The success of a spring embedder layout algorithm relies on the density of the graph more than it does on the number of nodes in the graph. For instance, even when there are a small number of nodes in a map, high connectivity in a small part of the map might make it impossible to successfully orient the edges in that part.

Figure 14 illustrates the fact that, some substrate and production nodes of a process node may be placed in another compound node (cellular location). During layout, the location of this compound node is determined with respect to the forces acting on it. Those additional forces may disrupt the proper orientation. This is a typical example, where multiple conflicting constraints are impossible to satisfy.

## 5 Conclusion

The main motivation behind this study was to build a specialized automated layout algorithm for PD maps that comply with the conventions in SBGN-PD maps. Our proposed algorithm adds the necessary heuristics to achieve this on top of a CoSE algorithm.

The first enhancement provides proper packing of complex members and disconnected molecules by using two different rectangle packing algorithms: tiling and polyomino packing. The second one supports port nodes and provides rotation ability for process nodes by introducing a new force type. An important point to note here is that, those enhancements are added without disturbing the force-directed structure of the algorithm. There is still room for improvement, however, especially in handling special cases such as irreversible processes.

Our proposed layout algorithm has been integrated into ChiLay library, which is also available through Paxtools.

## Supplementary Material

Supplementary Data
