# Supplementary material for: An algorithm for automated layout of process description maps drawn in SBGN
Source: Bioinformatics. 2015 Sep 10;32(1):77–84. doi: 10.1093/bioinformatics/btv516 (PMC4681988; doi:10.1093/bioinformatics/btv516)
Supplement: Supplementary Data [file supp_32_1_77__index.html]

An algorithm for automated layout of process description maps drawn in SBGN — An algorithm for automated layout of process description maps drawn in SBGN — Supplementary Data 

# An algorithm for automated layout of process description maps drawn in SBGN

## Supplementary Data

files

- Supplementary Data - docx file
