# Supplementary material for: An algorithm for automated layout of process description maps drawn in SBGN
Source: Bioinformatics. 2015 Sep 10;32(1):77–84. doi: 10.1093/bioinformatics/btv516 (PMC4681988; doi:10.1093/bioinformatics/btv516)
Supplement: Supplementary Data [file supp_btv516_SBGN-Layout-Bioinf-Supplementary.rev.docx]

# Supplementary Document for “An Algorithm for Automated Layout of Process Description Maps Drawn in SBGN”

### INTRODUCTION

As Figure 1 exhibits, layouts produced by general purpose graph layout algorithms fall short in certain significant ways. Our proposed layout algorithm is the only algorithm that successfully addresses these issues, producing layouts that comply with SBGN-PD notation (Figure 2).


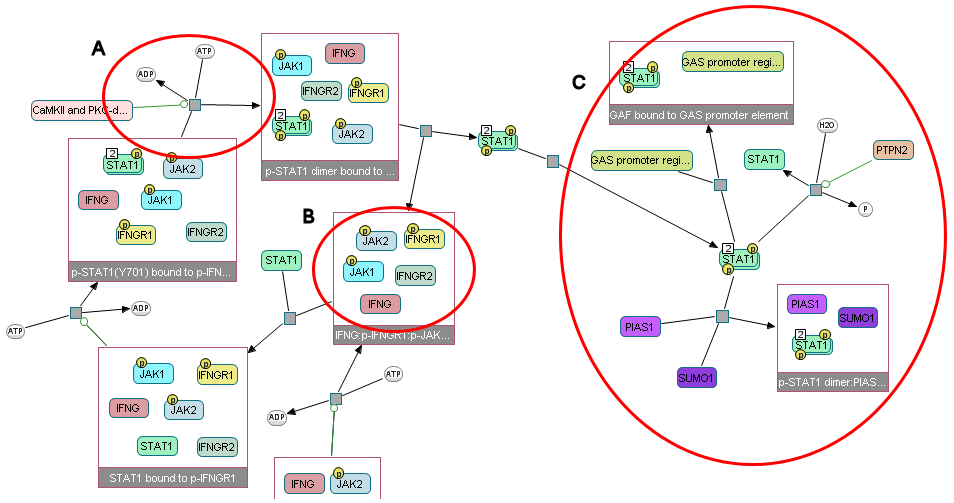


Figure 1 SBGN states that product and substrate edges of a process node (represented as small gray squares) should be placed on opposite sides of associated process nodes. Moreover, each process node should have two port nodes: an input and an output port. The substrates (products) of a process should be connected to the input (output) port (A). A general purpose layout algorithm will not properly pack degree zero members (represented as rounded rectangles with information bulbs) inside a molecular complex (B). The processes that take place inside a cellular compartment are not clearly separated from any other processes occurring outside that compartment (C).


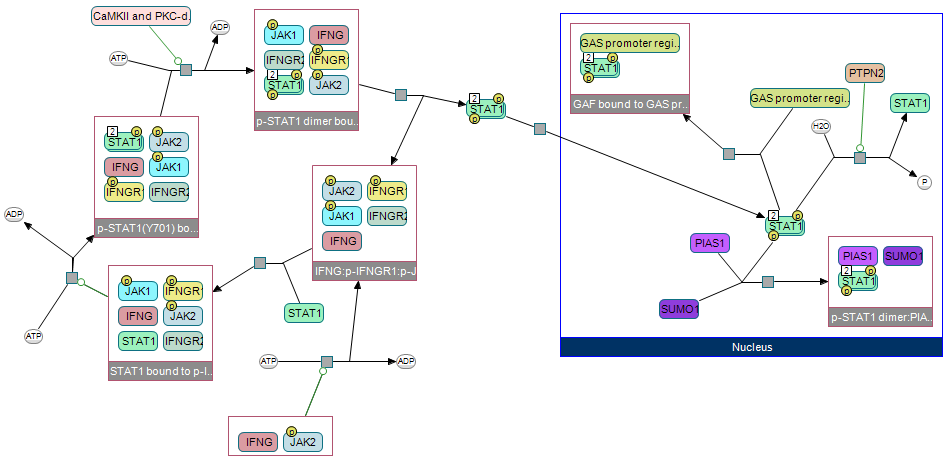


Figure 2 Proper SBGN layout of the biological diagram shown in Figure 1

### BACKGROUND

#### Graphs

A *graph* is a representation of a set of objects, called *nodes*, where some pairs of objects are connected by links, called *edges*. A node $v\in V$ (an edge $e\in E$), where $G=(V,E)$, is said to be a *member* of graph $G$; conversely, $G$ is said to be the $owner$ of node $v$ (edge $e$). A *directed graph* is one, where edges have a direction associated with them. A *directed acyclic graph* (dag) is a directed graph with no directed cycles. A *bipartite graph* is a graph whose vertices can be divided into two disjoint sets $U$ and $V$, where $U$ and $V$ are independent, such that every edge connects a vertex in $U$ to one in $V$. A *compound graph* $C=(V,E,F)$ consists of nodes $V$, adjacency edges $E$, and *inclusion edges* $F$ [1]. It is required that the inclusion graph $T=(V,F)$ is a rooted tree, and no adjacency edge connects a node to one of its descendants or ancestors.

#### Automated layout and CoSE

Force-directed layout algorithms (also known as spring embedders) are arguably the most popular type of automatic graph layout, where the basic idea is to simulate a physical system obeying the laws of Hooke and Coulomb. In such a system, nodes are assumed to be physical objects with a certain “electrical charge”, connected via “springs” of a pre-specified desired length. Objects pull or repel each other due to springs and electrical charges. The optimal layout is regarded as the state of this system, in which the total energy is minimal.

The formulas for calculating the two main types of forces in a spring embedder follow [1]. Spring force for an edge $e=(u,v)$ is

$\vec{F_{s}}=\frac{\left( \lambda-\left\| p_{u}-p_{v} \right\| \right)^{2}}{\eta}\vec{p_{u}p_{v}}$,

where $\lambda$ is the ideal edge length, $\eta$ is the elasticity constant of the edge, and $p_{u}$ and $p_{v}$ are positions of nodes $u$ and $v$, respectively.

Repulsion force between two nodes $u$ and $v$, on the other hand, is

$\vec{F_{r}}=\frac{\alpha}{\left\| p_{u}-p_{v} \right\|^{2}}\vec{p_{u}p_{v}}$,

where $\alpha$ is the repulsion constant.

Compound Spring Embedder (CoSE) is a force-directed layout algorithm that supports compound nodes [1]. Certain additions have been made on the basic spring embedder model to handle compound nodes. First, in order to handle varying node sizes (especially expanded nodes) and to avoid overlaps with neighboring nodes, calculation of edge lengths are based on the parts of edges in between the borders of end-nodes, as opposed to their centers [4]. Then, an expanded node and its associated nested graph are represented as a single entity, similar to a “cart”, which can move freely in orthogonal directions (no rotations allowed). Multiple levels of nesting is modeled with smaller carts on top of larger ones (Figure 3).


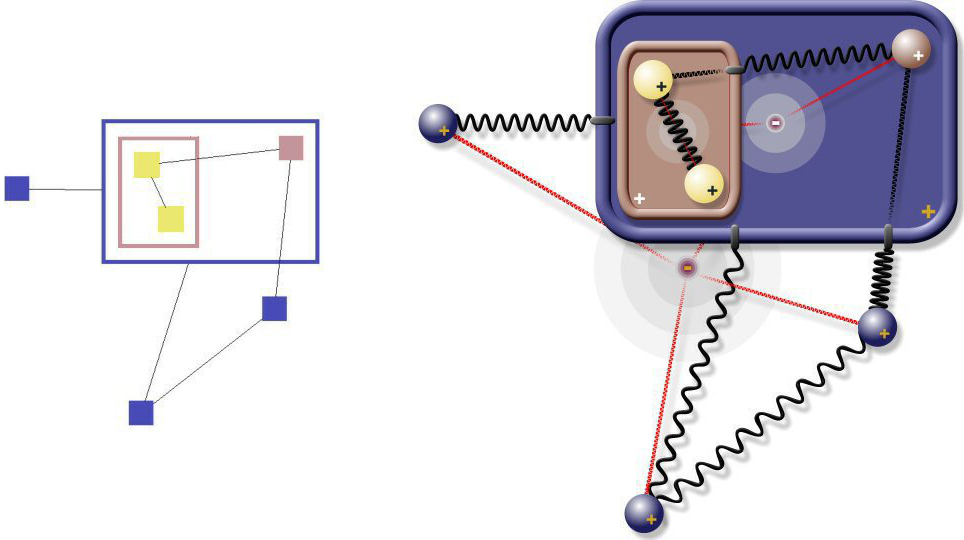


Figure 3 Part of a sample compound graph (left) and the corresponding physical model used by CoSE [1], where the deeper a node is in the nesting hierarchy the lighter it is colored (right).

### METHODS

#### Packing disconnected nodes

Packing can be integrated into SBGN-PD layout without any interference as a pre-processing step. For a molecular complex, its members are packed and the dimension of the molecular complex is set accordingly and its members are temporarily removed from the graph. In the case of disconnected nodes outside molecular complexes, dummy parent compounds are created. After the disconnected children of each dummy parent compound are packed, the size of the dummy compound is set accordingly and it's emptied before layout starts. After layout finishes, as a post-processing step, we simply insert temporarily removed members (children) back to its molecular complex (parent compound node). We finally remove any dummy compounds introduced temporarily (Figure 4).


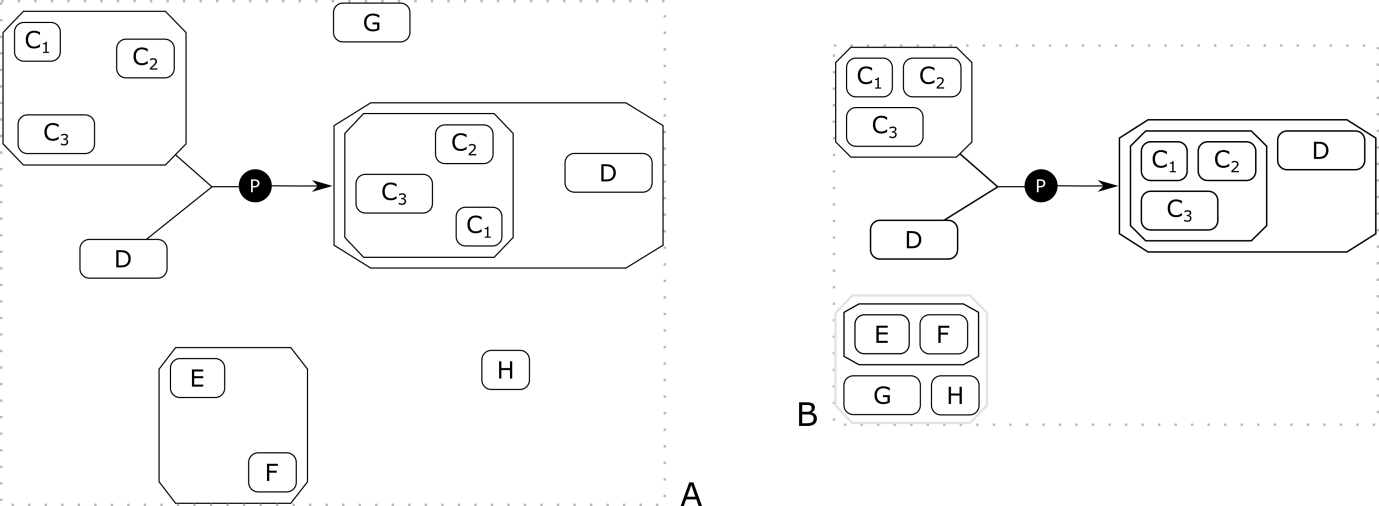


Figure 4 An example PD map containing many disconnected molecules laid out with a generic layout algorithm (A). The same map laid out using tiling to produce a more compact drawing, where dummy compound created for disconnected nodes outside of any molecular complexes and placed on lower-left corner is shown with gray bounds (B).

### IMPLEMENTATION AND RESULTS

#### Packing

For comparing tiling and polyomino packing methods, random compound graphs with no edges were generated using the following settings:

- *Minimum and maximum node dimensions*: (10 x 10) and (200 x 200), respectively.
- *Compound depth* (maximum depth of molecular complexes and cellular compartments): 3, since it is extremely rare to see more than 3 levels of nesting in SBGN-PD diagrams.
- *Number of siblings* (number of child nodes a compound can have): 5, since most compounds are molecular complexes with only a few members.
- *Branch factor* (probability of a branch getting pruned to randomize nesting depth): 0.4
- *Number of nodes*: 10, 20, 30, 40, 50, 60, 70, 80, 90, 100, 120, 150, 200, 300, 400, since most real PD maps used in interactive visualization are not huge. For larger graphs, we assume at least one complexity management technique was used [2].

Figure 5 compares the success of tiling and polyomino packing methods using their adjusted fullness as defined in the manuscript. Further compaction through visibility is usually of no use with tiling. More importantly, as can be seen from this plot, polyomino packing has a clear advantage over tiling with large number of nodes (>60) but for smaller graphs, like SBGN-PD maps, tiling performs just as well. Packing is run on relatively small number of nodes in the context of SBGN maps; hence, its running time is negligible. But as our tests confirm, tiling is significantly faster than polyomino packing in general (Figure 6).


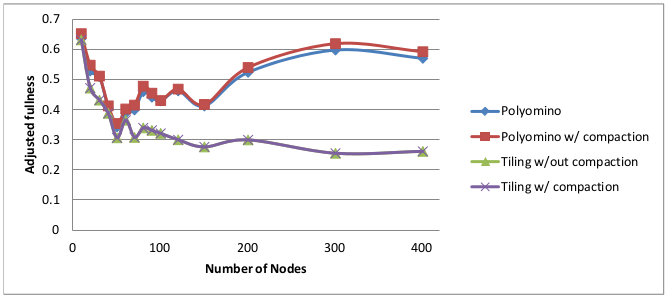


Figure 5 Quality comparison of tiling and polyomino packing with and without compaction.


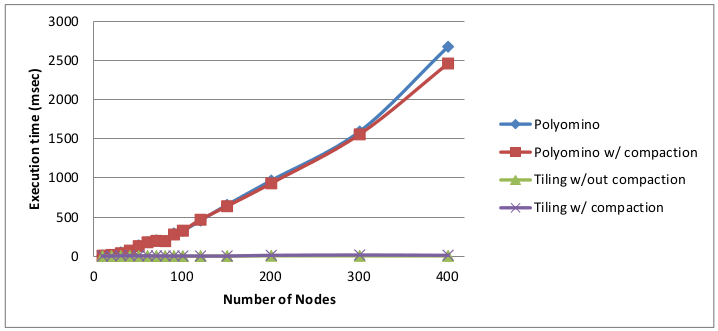


Figure 6 Run time comparison of tiling and polyomino packing with and without compaction.

#### Parameter tuning

The main criterion used for the success of the algorithm is the ratio of “properly oriented” edges to total number of edges in the graph. To decide when an edge is properly oriented, we use a parameter named *angle tolerance*. Thus, if the angle an edge $e$ makes with the edge at an ideal position is lower than angle tolerance, we say the edge $e$ is properly oriented. In our experiments, we take the value of this parameter as 100. Below are other parameters of our algorithm along with values that they are tested for:

- *Approximation distance* $ad=\{5, 20, 30, 50, 75, 100\}$: This parameter is used during location enhancement step as the radius of the circle in which a random position is chosen for a substrate, product or effector node, whose position is to be enhanced.
- *Approximation period* $ap=\{101, 211, 307, 401, 503, 701\}$: This parameter indicates the number of iterations between each application of the location enhancement heuristic. Prime numbers were used to eliminate the chance of finalizing layout right after application of the heuristic as we need at least a few iterations to stabilize approximated locations of nodes.
- *Rotation period* $rp=\{1, 2, 5, 10, 20, 30\}$: This parameter indicates the number of iterations between each application of the process rotation heuristic.
- *90-degree rotation force threshold* $c90=\{40, 60, 70, 90, 100\}$: This parameter indicates the minimum amount of total net force required for rotating a process node by 90 degrees.
- *180-degree rotation ratio threshold* $c180=\{0.4, 0.5, 0.6, 0.7, 0.8\}$: This parameter is used to decide whether or not a 180-degree rotation of a process node is to be performed. It defines a threshold for the percentage value calculated by dividing the number of obtuse-angled substrate and production edges to the total number of such edges.
- *Phase 1 maximum iteration count* $ip1=\{100, 200, 500, 1000, 1500\}$: This parameter is the maximum number of iterations performed in phase 1 of our algorithm, which is used to calculate a draft layout using the basic CoSE algorithm.

Before experimenting with individual parameters, we wanted to find the most coherent set of values of these parameters given a discrete set of values for each parameter as specified earlier. The best results are obtained as specified in Figure 7.


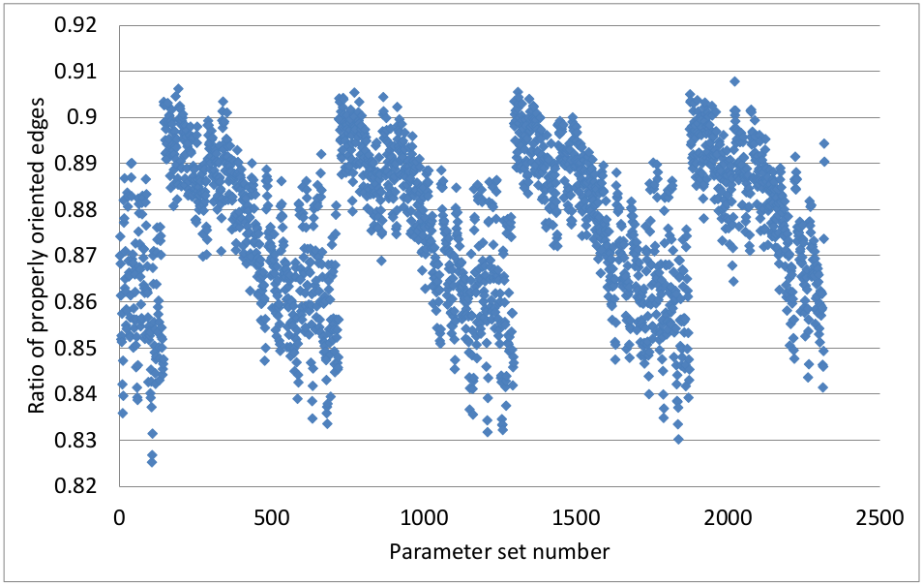


Figure 7 Experiments on most coherent parameter set. Simple search for a most coherent parameter set finds: $\boldsymbol{\{ad = 50, ap = 211, rp = 2, c}\boldsymbol{90 = 70, c}\boldsymbol{180 = 0.5, ip}\boldsymbol{1 = 200\}}$ with over 90 percent of edges properly oriented.

To confirm that changes in these parameters do not interfere with each other, we performed tests where only one parameter at a time was changed. The results can be found in Figure 8.

| 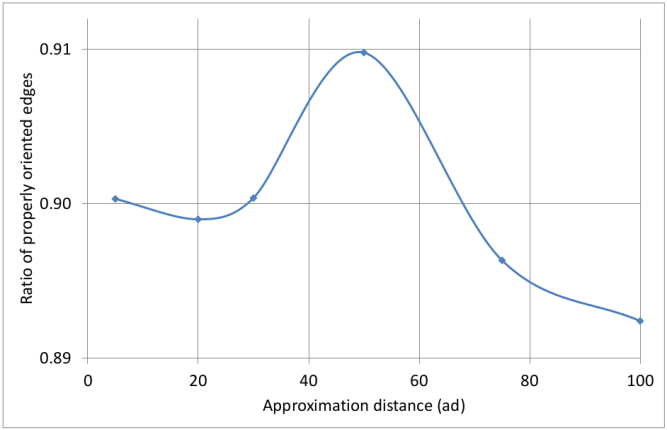 | 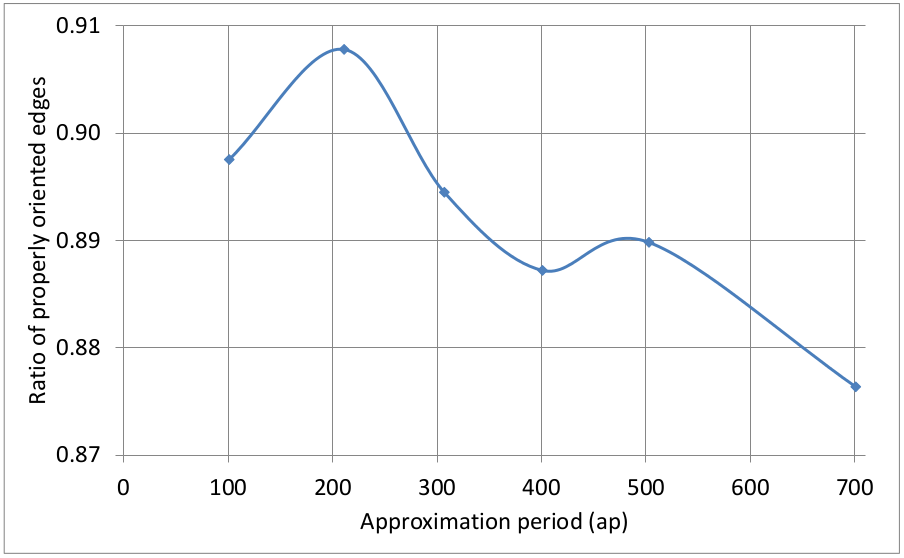 |
| --- | --- |
| 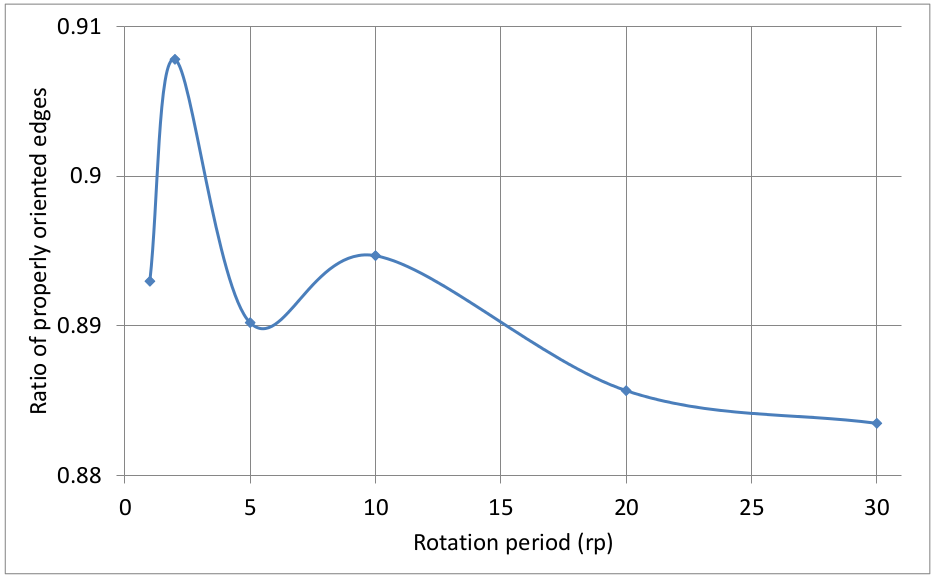 | 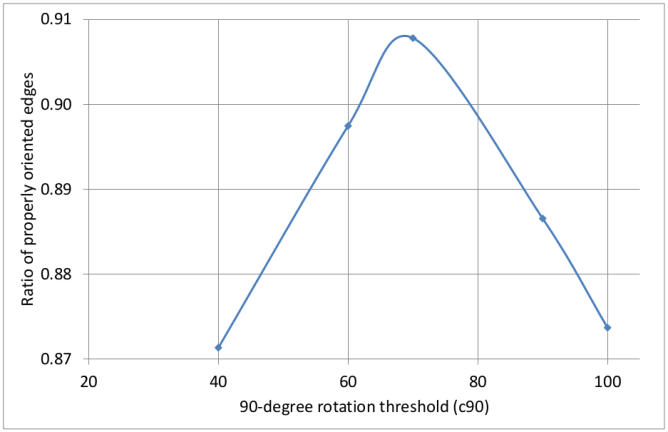 |
| 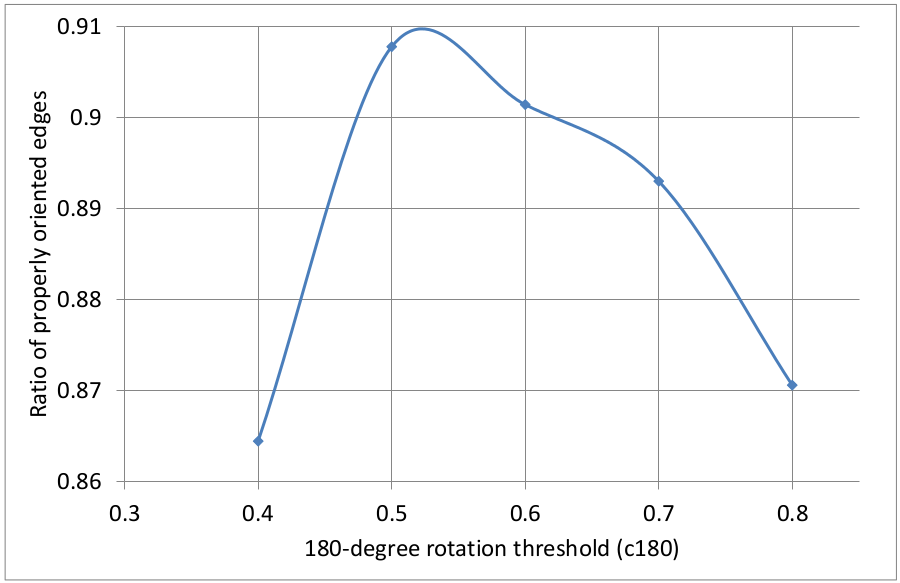 | 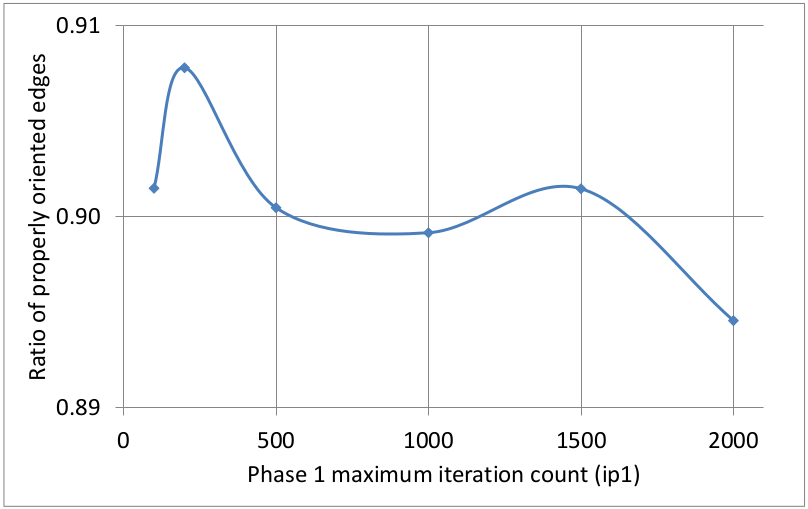 |

Figure 8 Effect of the change in an individual parameter on the success of the algorithm

#### Comparison with CoSE

Figure 9 through Figure 14 include some additional sample SBGN-PD drawings produced by our algorithm. Samples are taken from the Pathway Commons database [3] unless otherwise stated.


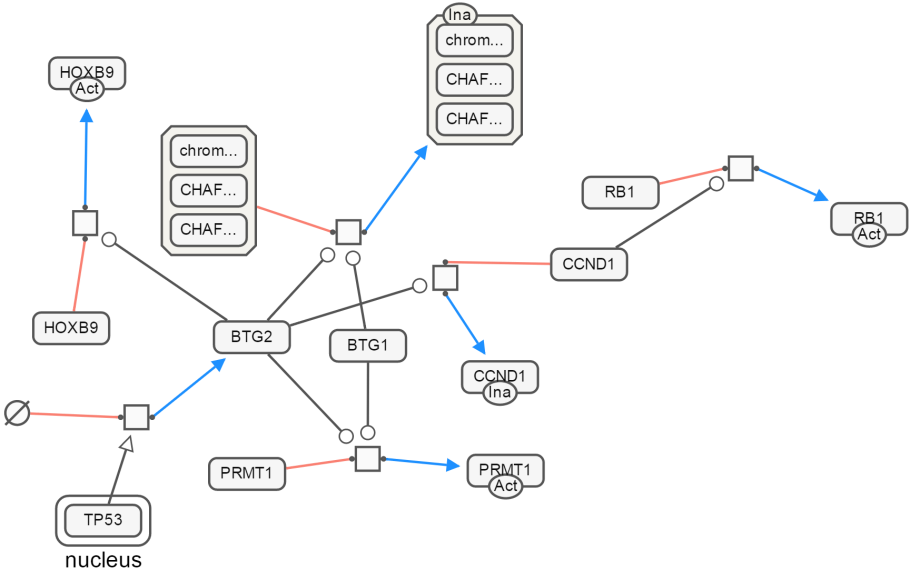


Figure 9 Btg family proteins and cell cycle regulation


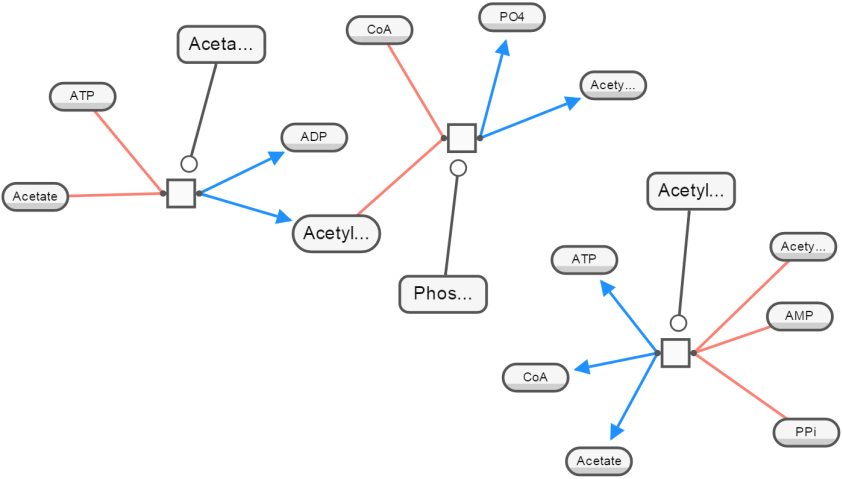


Figure 10 Acetate utilization [5]


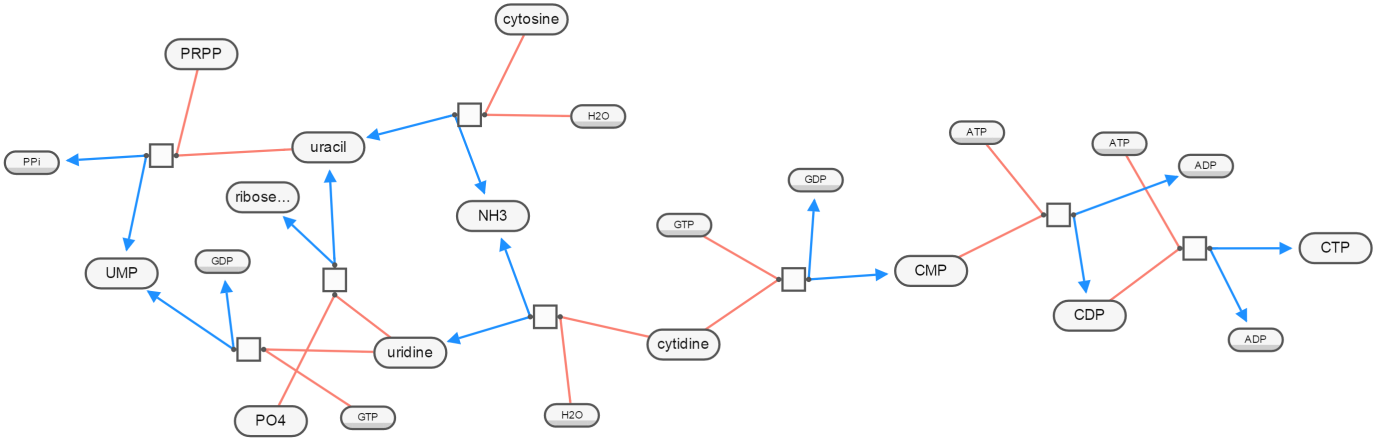


Figure 11 Salvage pyrimidine ribonucleotides [5]


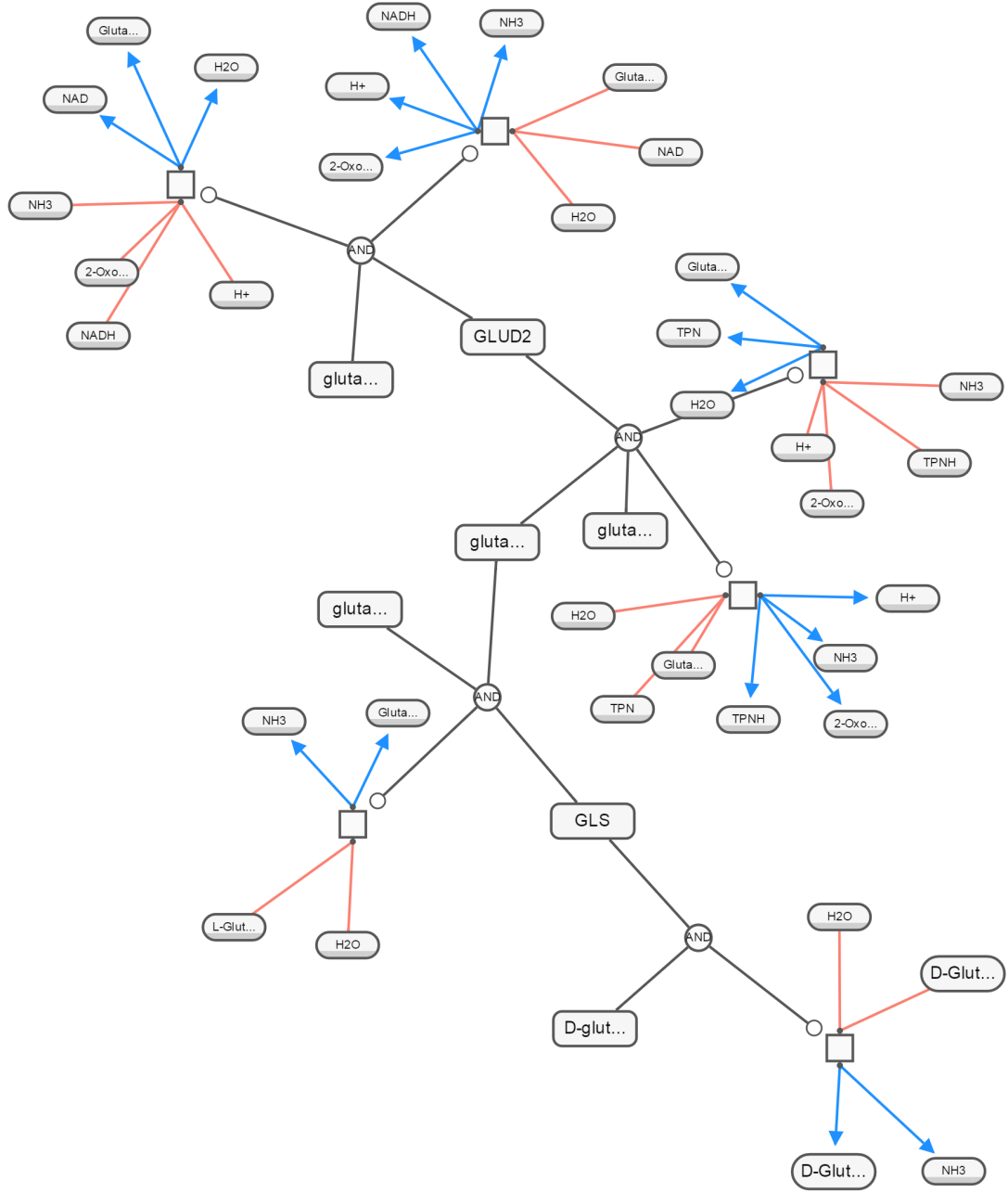


Figure 12 D-Glutamine and D-glutamate metabolism [6]


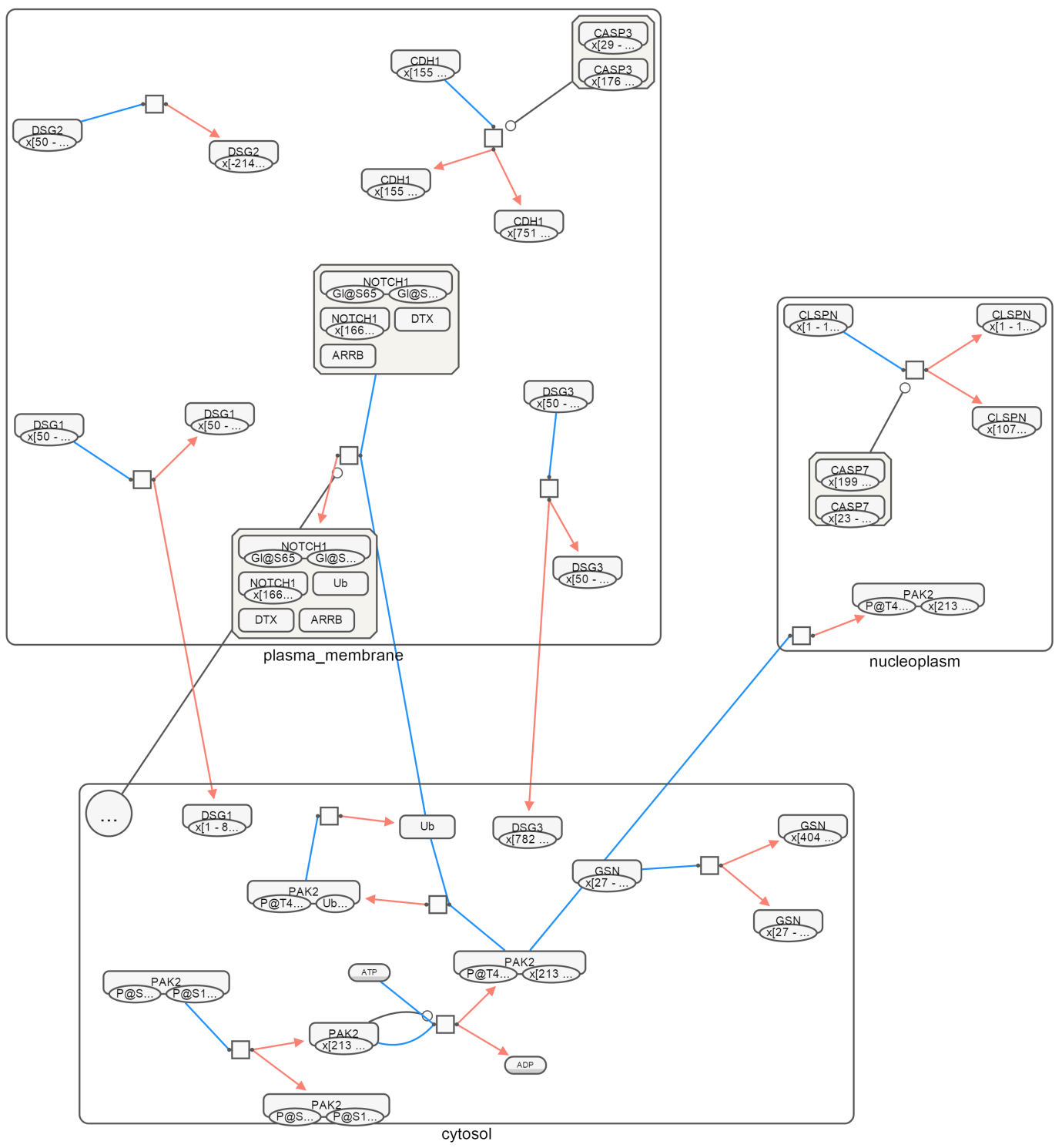


Figure 13 Activation of caspases through apoptosome-mediated cleavage


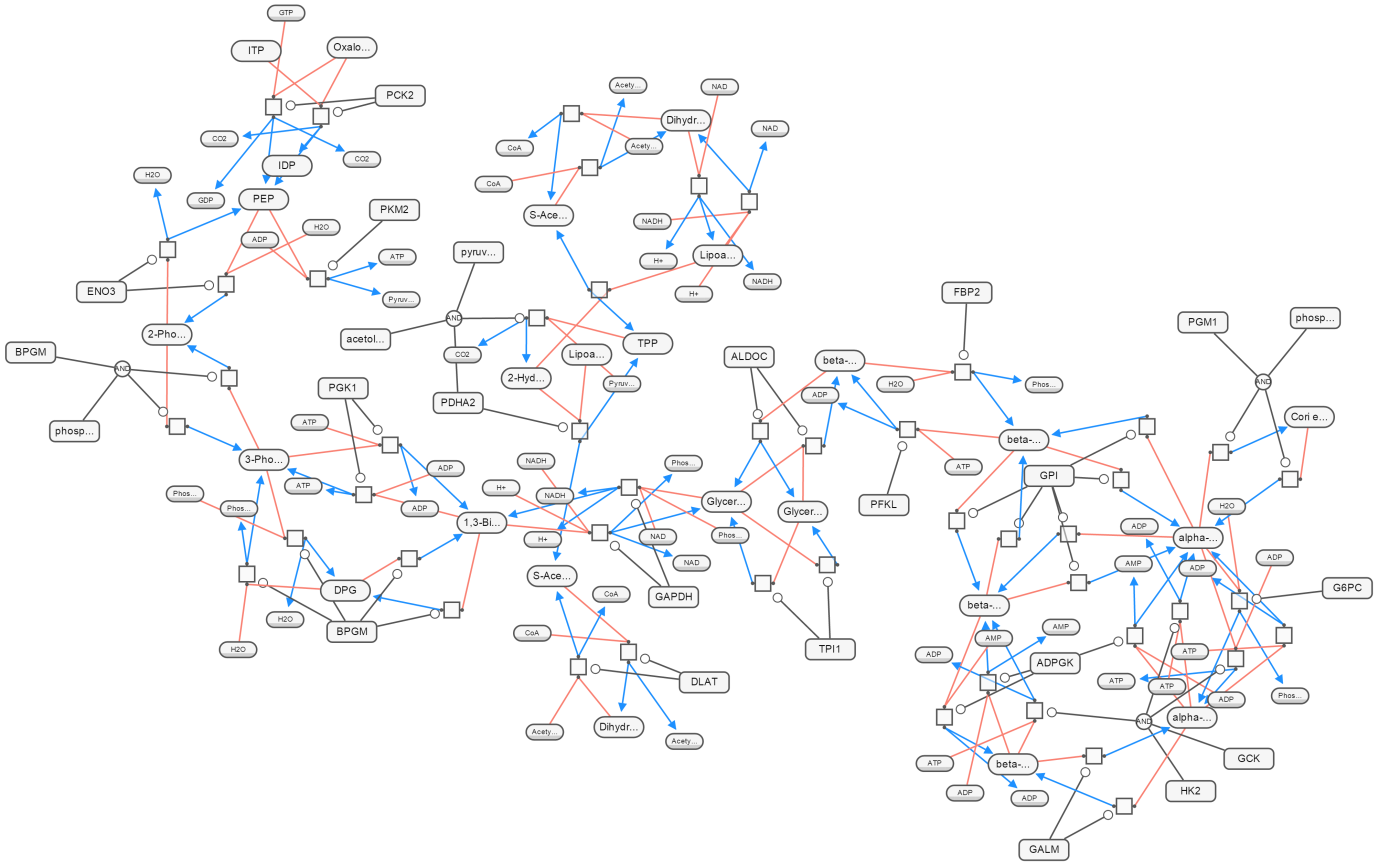


Figure 14 Glycolysis pathway

#### References

1. Dogrusoz, U. et al. (2009). A layout algorithm for undirected compound graphs. Information Sciences, 179, 980–994.
2. Dogrusoz, U. and Genc, B. (2006). A multi-graph approach to complexity management in interactive graph visualization. Computers & Graphics, 30(1), 86–97.
3. Cerami, E. et al. (2011). Pathway commons, a web resource for biological pathway data. Nucleic Acids Research, 39(suppl 1), D685–D690.
4. Harel, D. and Koren, Y. (2002). Drawing graphs with non-uniform vertices. In Working Conference on Advanced Visual Interfaces (Proc. AVI02), page 157-166. ACM Press.
5. Mi, H. et al. (2013). PANTHER in 2013: modeling the evolution of gene function, and other gene attributes, in the context of phylogenetic trees. Nucleic Acids Research, 41(Database issue), D377-D386.
6. Juty, N. et al. (2015). BioModels: Content, Features, Functionality and Use. CPT: Pharmacometrics & Systems Pharmacology, 4(2), 65-68.
